# A Case of Extra-Uterine Pregnancy

**Published:** 1879-05

**Authors:** Abner Hard


### ARTICLE IV.

A CASE OF EXTRA UTERINE PREGNANCY. Extract from a paper read before the Fox River Valley Medical Association, at Aurora, Ill., January 6, 1879. By ABNER HARD, M.D.

GENTLEMEN:—For presenting to your consideration the details of a case of extra uterine pregnancy which lately occurred in my practice, I think I need make no apology. It is an occurrence so rare that few country practitioners, and in fact few accoucheurs, have an opportunity to observe it.

Some years since, Doctor Allaire, a member of this society, had such an experience in his practice, where a German woman carried an extra-uterine fœtus. When the doctor was first summoned, there was a tumor in the side, whose sac inclosed fœtal bones, many of which were perfect, all the soft parts of the fœtus having been absorbed. The woman lived to have other children, and is living at this time, as we believe. The older members of the society will recollect the fact.

A case which came under the care of Drs. Winslow and Gillett, of this place, was diagnosticated as extra-uterine pregnancy, but, a post-mortem examination being refused them, they had no opportunity to verify their diagnosis. Believing so rare an occurrence should be put on record, I report my case.

Mrs. C., aged 36 years, had been married 16 years, but did not become pregnant until last spring. She was naturally very delicate, sensitive and nervous. On the 13th of June, 1878, I was called to see her, when suffering from uterine hæmorrhage. It was the time for her menses, but for several months she had been irregular in that particular, and I looked upon this as menorrhagia, though she had some symptoms of pregnancy, which she could not believe existed, and her pain simulated that of abortion. This condition yielded to treatment, and in a few days she was up again. July 9th, I was again summoned to attend her, when she complained as before, but also had congestion of the lungs, and unusual and constant pain in the abdomen. Her sufferings were excruciating. Relief was obtained only by the use of strong anodynes. At this time I became satisfied she was pregnant, and feared placenta prævia and an abortion, which the patient was very desirous of avoiding. She recovered and was about the house until July 28th, when I was again called, and found great tenderness over the abdomen, considerable nausea and dyspepsia. Very little food could be tolerated. She could get no sleep lying, and had to be kept in a semi-recumbent position. Careful examination failed to throw any additional light upon the case. The fœtal heart could not be heard, but the other usual signs of pregnancy were present. From this time until her death she was unable to go about the house. As the abdomen enlarged, the difficulty of breathing became great. Every movement of the diaphragm in breathing gave pain, and coughing was very distressing. The motions of the fœtus were feeble, but gave great pain; and added to her discomfort was incontinence of urine. Every month she menstruated; sometimes the flow was small, but accompanied with unusual pain. This condition continued increasing in severity until (as we reckoned) about the sixth month of gestation, from which time she felt no motion of the fœtus, and she grew worse more rapidly. Although there was no general peritonitis, the abdomen was so tender she could hardly bear light bed-clothing, and every attempt at digital examination *per vaginam* was resisted as unbearable. During most of the time her mind was greatly disturbed, not being fully rational. About ten days before her death there was an offensive discharge from the vagina, the odor resembling decomposed animal matter. Believing the fœtus to be dead and decomposing, and that death would soon ensue unless relieved of the dead fœtus, assisted by Dr. L. A. Winslow, on the 18th of November we gave her ether and made an examination which revealed the os uteri lying above the pubis and to the right of the median line, which could with difficulty be reached. She rallied from the effects of the examination and etherization, but continued to sink, and died Nov. 20th.

A post-mortem examination made seventeen hours after death, in presence of Drs. Robbins, Higgins, Grant, Murphy and Winslow, revealed a case of extra uterine pregnancy. The fœtus was plump and apparently well nourished up to the time of its death at about the sixth month. Decomposition had so far advanced that the skin came off very readily. Impregnation had taken place in the right ovary, from which the sac or membrane had grown which contained the child and secundines. Adhesions were very firm between the sac and peritoneum covering the intestines, and the wall of the sac was so fragile it was readily broken or torn by the finger. The liquor amnii was semi-sanguineous and offensive. The placenta was of natural size, partly decomposed and attached to the upper part of the sac. The uterus was small—not larger than a man's fist—and found lying above the pubis, to the right of the median line and in front of the fœtal envelope. Its mucous membrane was covered with a caseous or cheesy deposit about 0.2 Cm. in thickness, which was readily removed with the scalpel. The fallopian tubes appeared natural, the one leading to the right ovary being perfect its entire length, and as long as the left tube, showing, as I think, that this was a case of ovarian and not tubal pregnancy.

